# The Combined Use of Negative-Pressure Wound Therapy and Dermal Substitutes for Tissue Repair and Regeneration

**DOI:** 10.1155/2020/8824737

**Published:** 2020-12-04

**Authors:** Liping Zhang, Tingting Weng, Pan Wu, Qiong Li, Chunmao Han, Xingang Wang

**Affiliations:** ^1^Department of Burns & Wound Care Center, The Second Affiliated Hospital, Zhejiang University College of Medicine, Hangzhou 310009, China; ^2^College of Medicine, Zhejiang University, Hangzhou 310000, China

## Abstract

In clinical practice, skin defects occur frequently due to various kinds of acute and chronic diseases. The standard treatment for these wounds is autografting, which usually results in complications such as scar formation and new wounds at donor sites. The advent of dermal substitutes has provided a novel method for wound repair, and rapid angiogenesis of the dermal substitutes is crucial for the graft to take. At present, many strategies have been developed to improve the process of vascularisation, some of which have shown promising potentials, but they could be very far from clinical applications. Most recently, negative-pressure wound therapy (NPWT) has been used extensively in clinical practice for wound care and management. It has been reported that NPWT reduces the time required for vascular ingrowth into the dermal substitute and improves graft take, indicating great potentials for wound repair. This article presents a comprehensive overview of the combined use of NPWT and dermal substitutes for tissue repair and regeneration. Relative concerns and prospects are also discussed.

## 1. Introduction

**Figure 1:**
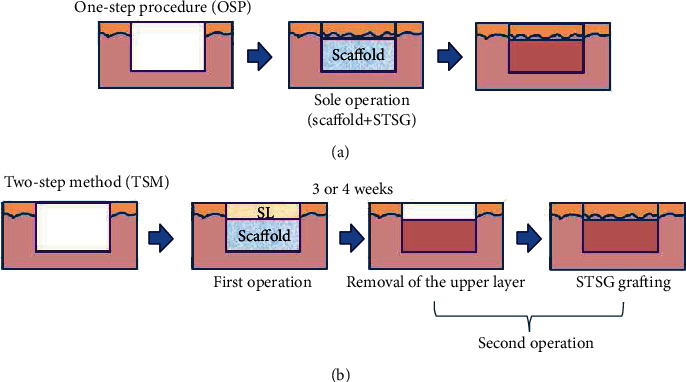
Two methods for the delivery of dermal substitutes to wounds. STSG: split-thickness skin graft; SL: silicone layer.

Severe skin defects resulting from various acute and chronic factors occur frequently in clinical practice. A traditional reconstruction ladder has been developed for wound repair and tissue reconstruction [1]. Autografts, particularly thin split-thickness skin autografts (STSGs), are the standard treatment for wound coverage [2, 3]. Long-term results usually include scar formation, tissue contracture, and other complications, as STSGs consist only of thin single layers of dermal tissue. Various flaps are often employed to reconstruct tissues at the recipient site for more complicated wounds characterised by bone or ligament exposure. However, new skin defects are usually created at the donor site. The advent of dermal substitutes has provided a novel method for the repair of various severe skin defects. These substitutes act as dermal regenerative templates, facilitating dermal reconstruction and regeneration. An ideal dermal substitute should provide a template with an appropriate three-dimensional (3D) porous structure and mechanical support to guide cell migration, extracellular matrix deposition, and angiogenesis [2]. Some commercially available dermal substitutes have been used widely to reconstruct wounds with one- and two-step procedures [4, 5]. The one-step procedure tends to involve skin defect closure through the simultaneous application of a dermal substitute and an STSG during one operation, but a nonvascularised dermal substitute may hamper graft survival (Figure 1(a)). The two-step procedure is utilised more frequently in clinical practice, although it requires a second operation to complete the repair process by transplant of an STSG (Figure 1(b)). Patients must wait 3–4 weeks before the second operation, to allow the dermal substitute to vascularise [4]. Rapid angiogenesis of the dermal substitute is very important for the graft to take. Various means of promoting dermal substitute vascularisation have the potential to improve the efficiency of wound repair and the quality of healing.

Methods developed to promote the vascularisation of engineered tissue include the incorporation of angiogenic factors [6–9], loading of functional genes into scaffolds [10, 11], and preseeding with vascular endothelial cells [12, 13]. All of these approaches have promising potential for rapid vascularisation, but they could be very far from clinical applications. Most recently, negative-pressure wound therapy (NPWT) has become more popular; it is now used extensively in clinical practice for wound care and management. NPWT plays an important role in wound repair and has many advantages, such as efficient drainage, reducing wound oedema, effectively controlling and reducing infection, promoting granulation tissue proliferation, and inducing cell growth [14].

The combined application of NPWT and dermal substitutes improves graft take [15, 16]. Recent evidence suggests that NPWT promotes the vascularisation of DS and simplifies the process of wound repair. NPWT enables DS to come into closer contact with the wound bed and shortens the transfer distance of nutrients and angiogenic growth factors. In addition, the combined application of NPWT and SD can reduce the risk of infection, effectively remove wound secretions and inflammatory factors, form physical pull on the wound edge through negative pressure, promote the proliferation of fibroblasts, and maintain the integrity of the neovascular network structure [17]. Some researchers have reported that NPWT reduces the time required for vascular ingrowth into the dermal substitute and improves graft take [15], indicating great potential for wound repair. This article presents a comprehensive overview of the combined use of NPWT and dermal substitutes for tissue repair and regeneration. The related mechanisms are also discussed.

## 2. The Roles of Dermal Substitutes in Wound Repair and Reconstruction

Dermal substitutes serve as dermal regenerative templates for neotissue ingrowth and deposition and have been developed and applied to restore severe skin defects in clinical practice [18]. Some commercially available substitutes, such as Integra®, Pelnac®, Matriderm®, and Alloderm®, have been used widely for wounds, burns, and orthopaedic applications, with one- and two-step surgical procedures [4, 5, 19]. Dermal substitutes recover the continuity and integrity of dermal tissue to some extent and provide porous 3D microstructures for the ingrowth of repair cells and newly formed blood vessels.

The two-step surgical procedure continues to be the method used more frequently in clinical practice (Figure 1(b)). For example, Integra®, a bilayer dermal substitute developed by Yannas et al. [20, 21], is a commercially available collagen-based dermal substitute combined with a silicone top layer that functions as a temporary epidermis to retain wound moisture and block microorganisms. The top silicone layer is removed and replaced by an STSG when the collagen-based layer appears to be vascularised. The two-step procedure seems to be essential to allow vascularisation of the scaffold and ensure a high STSG take rate. However, these dermal substitutes may take more than 3 weeks to become fully vascularised [22, 23]. During this period, the risk of wound infection becomes prominent. Patients who receive such treatment must undergo secondary operations to achieve complete wound closure.

The one-step procedure has become increasingly popular [24]. One-step grafting involves the simultaneous application of a dermal substitute and an STSG to close the wound in one operation (Figure 1(a)). This approach provides earlier wound closure and avoids the secondary operation, but the presence of a nonvascularised dermal substitute acts as a barrier to nutritional diffusion to the autograft. Hence, grafts are often lost. Before the complete vascularisation of the dermal substitute, the graft survives at the early stage only by diffusion of nutrients from the wound bed. Any barrier between the graft and wound bed inhibits this process, resulting in loss of the graft.

## 3. Mechanisms of NPWT

NPWT has many other aliases, such as VAC (vacuum-assisted closure) [25], the vacuum sealing technique [26], vacuum sealing drainage, topical negative pressure therapy [27], vacuum therapy, subatmospheric pressure therapy, subatmospheric pressure dressing, vacuum sealing, and foam suction dressing [28]. In 1993, the German surgeon Fleischmann first reported the use of this technique to treat wounds in open fractures; he found that NPWT provided efficient wound cleaning, marked proliferation of granulation tissue, and reduced morbidity from wound infection [29]. This technique requires the use of a specific foam or open-cell gauze to fill in the wound or tissue defect. A drainage tube with lateral holes is inserted into the specific foam. The foam and adjacent skin are covered with a transparent impermeable dressing. Connection of the drainage tube to a suction device, such as a vacuum bottle, produces negative pressure in the foam to drain the necrotic tissue or wound exudate [30, 31].

The concept of using negative pressure to create a suction force, enabling the drainage of wounds to promote healing, is well documented [32–35]. However, the precise mechanism underlying the effects of NPWT on wound healing is not known. Several mechanisms have been proposed, including removal of the surplus wound exudate and reduction of tissue oedema, regulation of local blood flow, inhibition of bacterial colonisation and prevention of cross infection, promotion of the proliferation of repair cells and inhibition of their apoptosis, and exertion of mechanical force to accelerate wound healing [36, 37].

### 3.1. Removal of Surplus Wound Exudates and Reduction of Tissue Oedema

The wound inflammatory reaction can result in oedema of the surrounding tissues and excess exudate. Tissue oedema upregulates the after-loading of capillaries and decreases the supply of oxygen, nutrients, and bioactive factors to the wound site. Wound fluid contains many proteases, which suppress angiogenesis and the proliferation of keratinocytes, fibroblasts, and endothelial cells [38]. Surgical drainage is one of the most basic techniques to expedite wound healing. By removing this protease-rich wound fluid, NPWT may decrease the levels of matrix metalloproteinases 1, 2, and 13; cathepsins; and elastases [25, 39], thereby regulating degradation of the extracellular matrix and promoting angiogenesis [40]. The removal of exudates also prevents the accumulation of inflammatory mediators and encourages the diffusion of nutrition into the wound. In addition, NPWT reduces oedema in surrounding tissues and decreases after-loading of the capillaries, thereby increasing local blood flow and tissue perfusion [36, 41].

### 3.2. Regulation of Local Blood Flow and Gradual Tissue Formation

Increased blood perfusion can have a positive impact on wound healing. Morykwas and coworkers [36] used needle-probe laser Doppler flowmetry to show that 125 mmHg sub-atmospheric pressure causes a four-fold increase in blood flow in a porcine excisional wound model. Chen et al. [42] investigated the effects of pressures of −5, −10, −15, and −20 kPa for 20 min at 10 min intervals on full-thickness excisional skin defects in rabbits. The results indicated that VAC promoted capillary blood flow velocity, increased capillary calibre and blood volume, stimulated endothelial proliferation and angiogenesis, narrowed endothelial spaces, and restored the integrity of the capillary basement.

### 3.3. Inhibition of Bacterial Colonisation and Prevention of Cross Infection

NPWT inhibits bacterial colonisation by accelerating low blood flow, increasing tissue oxygen tension, and enhancing neutrophil function and subsequent resistance to infection via the oxidative burst mechanism [41]. One study in which swine wounds inoculated with a human isolate of *Staphylococcus aureus* and a swine isolate of *Staphylococcus epidermidis* were treated with NPWT or a control moist saline dressing showed that NPWT significantly increased the elimination rate of bacteria compared with control wounds [36]. Human trials support such results, with bacterial counts from wounds subjected to NPWT decreasing from clinically significant levels to 10^2^–10^3^/g tissue [36]. Many other studies have confirmed these findings [43, 44]. However, a recent prospective randomised trial showed that nonfermentative Gram-negative bacilli decrease significantly, whereas *Staphylococcus aureus* increases significantly, in VAC-treated wounds, suggesting different effects of VAC on different kinds of bacteria [45]. In addition, bacterial infection has become a major factor responsible for perpetual inflammation and tissue destruction in nonhealing chronic wounds. Bacterial biofilms may have a significant role in this process [46]. NPWT compresses the biofilm architecture, with reductions in thickness and diffusion distance [47].

### 3.4. Promotion of Cell Proliferation and Inhibition of Apoptosis

Only those cells that stretch can divide and proliferate in response to soluble growth factors, whereas cells without stretch tend to undergo apoptosis [48, 49]. Cells are able to sense mechanical forces and respond by regulating specific genes and inducing cellular programmes. Potter et al. [41] seeded endothelial cells in vitro on four dermal substitutes with and without NPWT and found that the intermittent application of suction optimised endothelial cell invasion and increased graft angiogenesis and integration. Baldwin et al. [50] reported that negative pressure switches endothelial cells to a migratory and proliferative phenotype, and that intermittent, rather than continuous, negative pressure was beneficial for cell ingress, indicating an important potential proangiogenic mechanism by which NPWT promotes wound healing. Subatmospheric pressure, applied in an artificially closed space to partial-thickness burns, significantly decreases subburn cellular death [28]. A clinical trial conducted with eight animals investigated tissue biopsies taken from wound bases and edges before and after 5 days of NPWT. The authors found a 200% increase in endothelial cell proliferation by using immunohistochemical methods [51]. The application of micromechanical forces to wounds *in vivo* promotes wound healing through a cell shape-dependent mechanical control mechanism [52]. Furthermore, NPWT inhibits apoptosis of repair cells.

### 3.5. Provision of Mechanical Force to Accelerate Wound Healing

The basic concept of the participation of mechanical forces in the regulation of neotissue shape and growth is well known [53]. NPWT not only removes excess interstitial fluid, but also transmits mechanical forces to surrounding tissues, with resulting deformation of the extracellular matrix and cells. NPWT exerts a pulling/compressing force on the entire interface between the foam dressing and wound [41]. The foam distributes this centripetal force evenly and pulls the wound edges together, reducing wound size. The mechanical load generated permits vascularisation and tissue proliferation [54, 55]. Tractional forces stimulate cellular activity via transmembrane integrin adhesion complexes bound to the extracellular matrix, resulting in deformation of the cytoskeleton and release of secondary messengers, i.e., calcium ions, prostaglandins, inositol-triphosphate, and phosphokinase C [56, 57]. The application of NPWT generates local external forces that stimulate cells, such as fibroblasts and endothelial cells, by these mechanisms, resulting in tissue collagen production and angiogenesis, respectively [36, 44, 55]. The local micromechanical forces generated by VAC could be useful to stimulate wound healing by promoting cell division, angiogenesis, and the local elaboration of growth factors [52].

## 4. Applications of NPWT in Combination with Dermal Substitutes

### 4.1. NPWT plus the One-Step Method (Figure 2(a))

Dermal substitutes, which commonly lack vascular structure, can be applied in a one-step surgical procedure to repair full-thickness skin defects. In the early stage after transplantation, nutrition is supplied by simple diffusion from the wound bed and surrounding tissues. Subsequently, angiogenesis starts and takes at least 3 weeks to finish [23]. Simple diffusion is a low-efficiency way to supply nutrition and is easily affected by hematomas, seromas, infection, and dermal substitutes [58].

NPWT shortens the diffusion distance from the wound bed to the graft by mechanical forces and renews the wound exudate with its suction function. As local tissue oedema upregulates capillary after-loading and decreases the supply of oxygen, nutrients, and bioactive factors to the wound site, NPWT removes the surplus wound exudates and reduces tissue oedema [36, 37]. In addition, NPWT regulates local blood flow and inhibits bacterial colonisation. Taken together, NPWT has the potential to improve the nutritional supply to a wound site and to promote wound healing.

Rapid angiogenesis of dermal substitutes is also very important for graft take. NPWT has been reported to have the potential to promote cell proliferation, penetration, and angiogenesis [51]. Molnar et al. [59] used NPWT to manage Integra®-treated complex tissue defects. The Integra® take rate was 96%, and STSG grafting was performed at 4–11 days, with a 93% take rate, indicating that NPWT improved the take rate and time to vascularisation of Integra®. However, a prospective study conducted by Moiemen et al. [60] showed that the application of NPWT dressings to dermal templates reduces shearing forces, restricts seroma and haematoma formation, simplifies wound care, and improves patient tolerance, but it did not demonstrate that NPWT accelerates neovascularisation as verified by the presence of histologically patent vascular channels. Hence, the level of angiogenesis cannot be related to the improved take of the autograft in the one-step procedure. The way in which nutrients diffuse is very critical for graft take before complete vascularisation of the dermal substitute. In the early stage of transplantation following the one-step surgical procedure, NPWT compromises the barrier resulting from the existing dermal substitute by altering the nutritional supply. Moreover, NPWT improves the take of autografts by establishing a rapid balance between the nutritional supply to the wound bed and the nutritional requirement of the graft in the relatively closed moist environment.

### 4.2. NPWT plus the Two-Step Method (Figure 2(b))

Skin defects can be repaired effectively using the two-step method of dermal substitute and then STSG application. NPWT is also used to manage wounds receiving the transplanted dermal substitutes in the two-step surgical procedure. NPWT promotes vascularisation of the dermal substitutes. When the dermal layer becomes well vascularised, a wound can be repaired further by the transplantation of cultured epidermal film or an STSG, with a high degree of efficiency.

The main basis of the use of NPWT plus the two-step method is that it promotes wound healing by NPWT to accelerate rapid vascularisation of the dermal substitute, but findings regarding the clinical efficacy of this approach differ markedly. For example, a retrospective study conducted by Moiemen et al. [60] showed that NPWT does not accelerate vascularisation of the dermal substitute. The proportions of neovascularisation in the dermal substitute were 0%, 20%, 60%, and 81% at 7, 14, 21 and 28 days, respectively, after dermal substitute transplantation. The average interval between the two operations was 33 days, and the average survival rate of the skin graft was 98%. In contrast, Jeschke et al. [16] showed that NPWT accelerates vascularisation of the dermal substitute. Compared with the control group, the NPWT group in that study had significantly shorter intervals between the two operations (24 vs. 10 days) and a significantly higher skin graft survival rate (78% vs. 98%). However, fibrin gel was added in the NPWT group, which affected judgment of the therapeutic effect of NPWT. Molnar et al. [59] used NPWT plus the two-step method to treat complex wounds and found that the interval between surgeries could be shortened to 4–11 days (mean, 7.25 days). The average survival rate of dermal substitutes is 93%, and that of skin grafting is 96%.

## 5. NPWT Parameters and the Vascularisation of Dermal Substitutes

### 5.1. Value of Negative Pressure

Argenta et al. and Morykwas et al. [25, 36] demonstrated that a negative pressure of 125 mmHg maximises blood flow to invasive and basal peritoneum in an animal experiment. Therefore, this pressure is used widely as a clinical standard for NPWT. However, whether it should be used for some special wounds, such as diabetic foot wounds, arterial ulcers, and other ischemic wounds, or when the negative pressure dressing covers a large area. Optimised negative pressure values could thus differ for wounds receiving transplanted dermal substitutes via the one- and two-step methods.

### 5.2. NPWT Operation Model

Negative pressure modes are generally of many types, such as continuous, intermittent, and sinusoidal wave. Potter et al. [41] suggested that compared with continuous vacuum suction, intermittent vacuum suction accelerates the migration of endothelial cells in dermal substitutes and stimulates a stronger vasodilator response. The sponge-like porous structure of Integra™ is more conducive to the transmission of negative pressure. Baldwin et al. [50] also showed that intermittent NPWT is more conducive to the endothelial cell migration and proliferation phenotype than persistent NPWT, indicating that NPWT promotes wound healing by accelerating vascularisation. The incorporation of a dermal substitute between the negative pressure foam and the wound bed potentially changes the distribution of mechanical forces provided by NPWT and can change the operation mode of NPWT, although this effect has not been demonstrated.

## 6. Issues and Prospects

The efficacy of NPWT plus dermal substitute use is controversial. Although numerous clinical studies have shown that NPWT promotes the vascularisation of dermal substitutes, some experts have expressed different opinions. For example, Greenhalgh [58] questioned the results of Bloemen et al. [15] and stated that dermal substitutes could impede nutrient supply to transplanted autologous skin before unvascularisation in the one-step procedure, leading to the failure of the skin graft. Greenhalgh [58] pointed out that the wounds selected in the study were small, and not all were third-degree wounds. Therefore, large autologous skin grafting is a better choice in terms of the length of stay, skin graft survival rate, and suppression of long-term scarring.

The economic benefits of this treatment are also controversial, although a study conducted by Hop et al. [61] showed no significant difference between the NPWT plus dermal substitute group and other treatment groups in the cost of early wound repair and later (long term) scar treatment. The two recognised mechanisms of angiogenesis are angiogenesis and vascularisation [33]. Neovascularisation is the route of nutrient delivery, but in the early stages of the one-step procedure, transplanted epidermal nutrition is available before the dermal substitute becomes vascularised. The supply is suppressed. Considering that NPWT shortens the dispersion distance between the wound bed and the transplanted epidermis, it facilitates the timely aspiration of wound exudates, reduction of oedema, improvement of local blood flow, and suppression of bacterial colonisation.

NPWT results in rapid balancing of the nutrient supply to the wound bed. However, this balance can be affected by the negative pressure and whether it is intermittent or continuous. Wang et al. [62] proposed the “simple diffusion” hypothesis for nutrients. They reported that NPWT creates a rapid balance between the nutrient supply to the wound bed and the nutritional requirements of the graft in the relatively closed moist environment, improving autograft take. However, this balance can be affected by negative pressure and whether it is intermittent or continuous. Dermal substitutes, which lack a vascular structure, can be applied in a one-step surgical procedure to repair full-thickness skin defects. In the early stage of transplantation, before the dermal substitute is fully vascularised, the main nutrient supply is via simple diffusion from the wound bed and surrounding tissues.

In many studies, meshed or punched dermal substitutes were employed in the NPWT plus one-step method and NPWT plus two-step method to facilitate drainage and avoid accumulation of the exudate under the dermal substitute. Obviously, existing punches or meshes in the dermal substitute can also facilitate ingrowth of neotissue and delivery of nutrition, which potentially improve the take of transplanted STSGs. Accumulating evidence indicates that NPWT has the potential to promote vascularisation and simplify the process of wound repair. However, little evidence is available from randomised controlled trials and comparative clinical studies, resulting in the low strength of evidence supporting the positive role of NPWT in promoting vascularisation. Some data from animal models and experiments indicate the possible dynamic mechanisms of NPWT in the regulation of the take of dermal substitutes; however, some controversies, including those regarding the process of vascularisation, the delivery of nutrition and oxygen, and healing quality, remain. All of these issues require more study to provide more data. In addition, some NPWT parameters, such as the value of negative pressure, operation modes, foam materials, and use or nonuse swilling, could play important roles in the vascularisation of dermal substitutes. The optimisation of all of these parameters for the combined application of NPWT and dermal substitutes should also be considered.

## Figures and Tables

**Figure 2:**
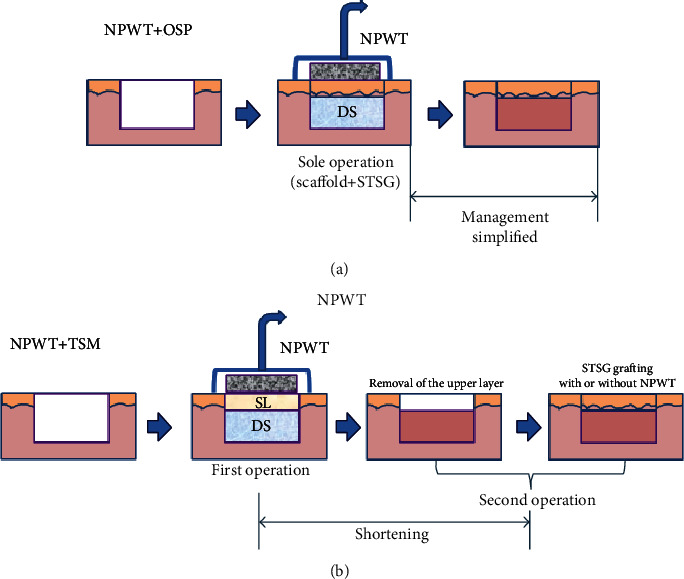
The combined use of NPWT and dermal substitutes in one-step and two-step procedures. NPWT: negative-pressure wound therapy; OSP: one-step procedure; DS: dermal substitute; STSG: split-thickness skin graft; TSM: two-step method; SL: silicone layer.
